# Real-time volume rendering for three-dimensional fetal ultrasound using volumetric photon mapping

**DOI:** 10.1186/s42492-024-00177-4

**Published:** 2024-10-25

**Authors:** Jing Zou, Jing Qin

**Affiliations:** Centre for Smart Health, School of Nursing, the Hong Kong Polytechnic University, Hong Kong, China

**Keywords:** Three-dimensional fetal ultrasound, Volume rendering, Photon mapping, Global illumination

## Abstract

Three-dimensional (3D) fetal ultrasound has been widely used in prenatal examinations. Realistic and real-time volumetric ultrasound volume rendering can enhance the effectiveness of diagnoses and assist obstetricians and pregnant mothers in communicating. However, this remains a challenging task because (1) there is a large amount of speckle noise in ultrasound images and (2) ultrasound images usually have low contrasts, making it difficult to distinguish different tissues and organs. However, traditional local-illumination-based methods do not achieve satisfactory results. This real-time requirement makes the task increasingly challenging. This study presents a novel real-time volume-rendering method equipped with a global illumination model for 3D fetal ultrasound visualization. This method can render direct illumination and indirect illumination separately by calculating single scattering and multiple scattering radiances, respectively. The indirect illumination effect was simulated using volumetric photon mapping. Calculating each photon’s brightness is proposed using a novel screen-space destiny estimation to avoid complicated storage structures and accelerate computation. This study proposes a high dynamic range approach to address the issue of fetal skin with a dynamic range exceeding that of the display device. Experiments show that our technology, compared to conventional methodologies, can generate realistic rendering results with far more depth information.

## Introduction

Fetal ultrasound is a reliable, practical, cost-effective, and efficient imaging method that captures images of a developing fetus using sound waves. Obstetricians can monitor pregnancy and assess the baby’s development using fetal ultrasound images. In certain cases, fetal ultrasonography can be used to diagnose potential issues, allowing obstetricians to intervene appropriately and quickly. Three-dimensional (3D) ultrasonic technology can provide high-resolution fetus reconstruction images quickly and accurately. Rendering 3D ultrasound can provide a more realistic visualization, allowing obstetricians to assess the growth of the fetus and make diagnoses more easily and accurately.

Most ultrasonic rendering approaches have been developed based on rendering techniques that use local illumination algorithms [1], which allow for simple visualization of 3D data and are ineffective at differentiating between different tissues and organs for examination and diagnosis. In addition, local illumination only generates a plastic-like visualization and cannot simulate the real lighting effects of human tissues. More critically, speckle noise affects typical ultrasonic data, and since the normal vector modules for local illumination rely on noise-sensitive gradient magnitudes of scalars, the resulting outputs are unsatisfactory. Additionally, compared with CT or MRI data, the dynamic intensity range of the ultrasonic images was significantly lower, rendering results, which were poor in terms of visual perception.

The visual impression of the rendered results can be improved using advanced illumination models, ambient occlusion (AO) [2, 3] and slice-based rendering algorithms [4, 5] that can imitate the scattering effect and produce realistic rendering results. The scattering effect describes the radiance energy of light, whereas a single scattering (SS) effect refers to scattering only once before reaching the viewpoint and a multiple scattering (MS) effect depicts multiple time scattering. Following earlier publications, we viewed the shade from the SS as a direct illumination effect and the shading from the MS as an indirect illumination effect. The technique used to approximate the MS effect is the volumetric photon mapping (VPM) technique [6, 7]. It is challenging to meet interactive and real-time requirements when using VPM to produce 3D fetal ultrasound during examinations. Therefore, it is necessary to balance rendering performance and visual quality.

During a routine prenatal examination, when a clinician adjusts the light source position or rotates the fetus, they must redesign the transfer functions to achieve optimal rendering results. Thus, the rendering system should provide interactive and timely visual feedback during examinations. In addition, speckle noise commonly degrades ultrasonic data, significantly lowering the picture signal-to-noise ratio. A system should tolerate the noisy nature of ultrasound imaging and improve the perceptual quality of the images. To fulfill these objectives, we propose a realistic real-time volume-rendering system for 3D fetal ultrasound. In this system, direct and indirect illuminations are rendered individually, and the effects of indirect illumination are computed using VPM. For efficiency, we employed a volume texture to store the spatial positions of the photons and flux. In addition, to achieve real-time rendering, a new screen-space radiance estimation method was suggested. Our contributions can be summarized as follows:

- First, we develop a realistic, efficient system for rendering of 3D ultrasound fetal data to improve visual impression with anti-noise ability.
- Both direct and indirect illuminations are taken into consideration for the estimation of global illumination effects. To speed up rendering, they are displayed independently by storing them in illumination volumes.
- We suggest a screen-space method that renders illumination in real time to estimate each photon’s radiance.

### 3D ultrasound rendering

There are always small white and black dots (known as speckles) in ultrasonic images. Therefore, noise removal is important in 3D ultrasound visualization. Previous studies on 3D ultrasonic rendering focused on rendering a smoother surface using appropriate transfer functions for noise removal. Various adaptive filtering algorithms [8–10] have been proposed as preprocessing steps for ultrasonic images. To accelerate the 3D noise filtering, a 3D mipmap-based noise reduction technique [11] was proposed. Recently, a fast 3D filtering method [12] using parallel bilateral filtering with the ability to adaptively change the kernel window was proposed. The region of interest is occluded by other surrounding tissues in 3D ultrasound. To remove this unwanted data, an interactive editing method was proposed [13]. For spatial and temporal 3D ultrasonic calibration, an image-based method [14] for freehand ultrasound systems was proposed.

Levoy [1] proposed a rendering technique termed “ray-casting” for visualizing medical volume data, including ultrasound. Rays were cast from the pixels in the output image into a volume. Volume classification and shading are the two main processes used in rendering techniques. Volume classification, also known as transfer function design, involves choosing a voxel’s visibility based on whether it is part of the user’s area of interest. To highlight the tissue-fluid boundaries, Honigmann et al. [15] proposed a technique for the adaptive design of opacity transfer functions. Their proposed method is also appropriate for 4D imaging of time-varying volume datasets [16]. Lim et al. [17] introduced a volume-rendering framework based on ray casting that filters ultrasonic data using programmable fragment shader operations. In ultrasonography, other rendering methods such as splatting [18] are also applicable, and a different opacity classification is suggested to depict smooth surfaces; however, these rendering techniques are not as high quality as ray casting. Few studies have been published on methodologies for rendering ultrasound data.

### Global illumination

In addition to volume classification, another important operation in volume rendering is shading through a local or global illumination model. Local illumination algorithms have been used extensively for volume rendering. Rather than employing surface normals, they utilized the gradient magnitudes of the intensity scalars [1] to assess diffuse and specular reflections. Global illumination can improve spatial perception and produce more realistic results. However, using global illumination effects in interactive volume-rendering systems is costly. Approximation techniques have been proposed to address this problem, as follows: For a more thorough overview of earlier studies, readers can refer to refs. [19, 20].

Half-angle slicing is the most well-known slice-based technique [21] that produces scattering effects and volume shadows [4]. The directional occlusion shading method [5] was suggested by Mathias (2012) to solve the issue of the slicing axis’ adaption to the location of the light. Previously, Schott et al. [22, 23] introduced the interaction between the geometry of light and volumetric participate media, which allows the integration of the geometry. Multiresolution volume storage was also utilized to accelerate rendering. Similarly, a Monte Carlo ray tracing technique based on a GPU was recommended by ref. [24] to produce simple scattering and AO for isosurfaces. A volumetric light technique that satisfies the interactivity criteria was proposed by Ropinski et al. [25], and ray casting was used to produce the final volume with acceptable quality. Kwon et al. [26] introduced a novel global illumination technique that used calibrated lighting aligned with the progression direction tailored for volume-ray casting in non-Cartesian coordinates. In addition, they optimized the lighting process by employing a light-distribution template, which significantly reduced the computational overhead associated with lighting operations. Yuan et al. [27] developed a cinematic volume-rendering algorithm based on photon mapping that supports multilight illumination. This algorithm effectively enhances the perception of depth and shape in the region of interest when influenced by multiple light sources. Moreover, Zhang and Ma [28] introduced a technique that can simulate light propagation by employing a convection-diffusion equation. The diffusion component of the equation describes how indirect light spreads inside the volume, whereas the convection component shows how direct light radiates in a volumetric medium. However, using volume shading techniques to interactively address general scattering effects is unrealistic. By contrast, our technique can provide various volumetric global illumination effects in real-time.

### Photon mapping

Owing to its excellent efficiency and adaptability in handling various lighting effects, photon mapping is commonly used for global illumination. The common photon mapping algorithm developed by Jensen [29] includes photon-tracing and rendering steps. In the initial step, photons from the light sources are emitted through the volume using a Markov random approach [29]. A k-dimensional (KD) tree was used to store the photon’s position, incidence direction, and power on the photon map. Photons can pass through each sample point undisturbed or interact with it (absorbed or scattered). The phase function, also known as the bidirectional reflectance distribution function, is adopted to establish a new direction for the photon if it is scattered. The in-scattered radiance was calculated in the subsequent step by seeking the K-nearest neighbor photons.

Increasing the efficiency of the conventional photon mapping approach, Havran et al. [30] introduced reverse photon mapping to speed up the calculations at the last collection stage. In ref. [31], Czuczor et al. proposed the storage of photon collision information in GPU texture memory. In addition, a texture filtering method was used in place of the conventional nearby-seeking approach based on the KD tree structure. However, this method is only effective for surface rendering. A real-time KD tree was first proposed by Zhou et al. [32] and built using GPU programming. KD tree nodes were constructed using this technique in real-time using a breadth-first search. A hierarchical photon mapping approach was proposed by Spencer and Jones [33]. This method increases the accuracy of illumination computation by passing photon energy layer-by-layer through the system, starting at the base of the binary tree and ending at the top layer. Progressive photon mapping was developed by Hachisuka et al. [34], who used a multipass rendering technique and continuously emitted photons into the image to gradually reduce inaccuracy. The photon mapping method was recently modified to produce an interactive character for real-time realistic volume rendering. To allow effective updates to the photon map, Daniel et al. [35] created a data format for assessing the scene areas affected by altering the transfer function. The volumetric photon mapping algorithm was incorporated into the customary predesigned radiance transfer procedure in a study by Zhang et al. [6].

## Methods

**Fig. 1:**
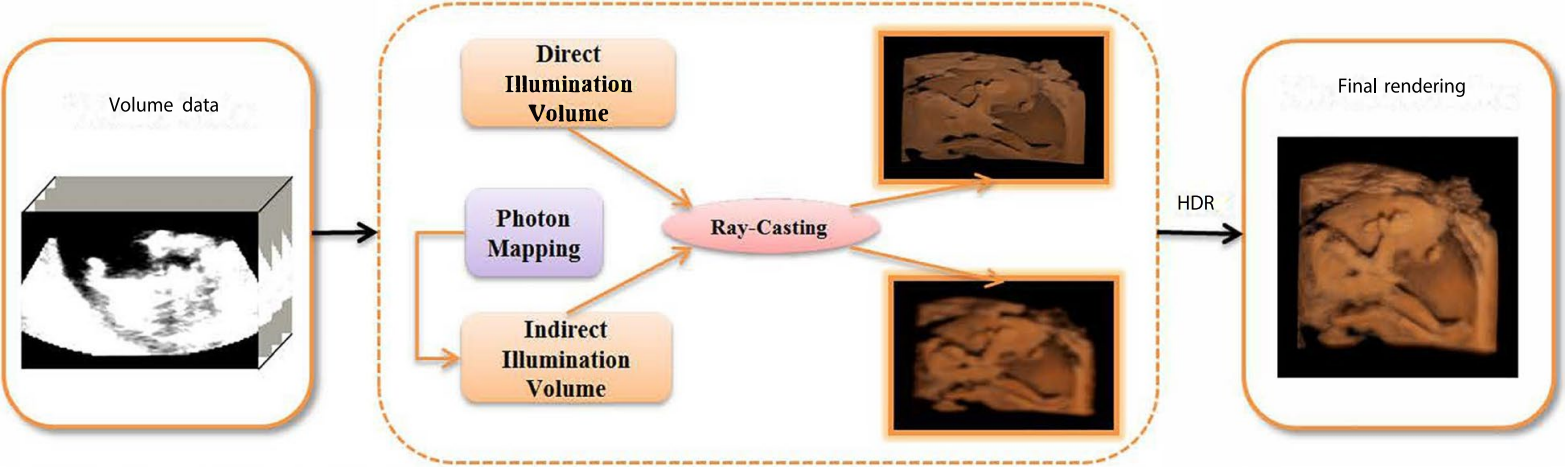
Work pipeline of our proposed method

In this section, we discuss the rendering process in detail. The workflow of the proposed method is illustrated in Fig. 1. Direct and indirect lighting were rendered separately throughout the rendering process, beginning with a raycaster. The rendering results for these two illuminations are shown in the middle box of Fig. 1. The details, particularly our indirect illumination computation method, are presented in Direct illumination and Indirect illumination subsections. To tackle the wide range of intensities resulting from varying lighting conditions, we propose a high dynamic range (HDR) technique.

When rendering the ultrasound data, emissions were not considered. Subsequently, Eq. 1 can be written as follows:

1 $$\begin{aligned} L(x_{s},\omega _{0}) & =L_{0}(x_{0},\omega _{0})T(x_{0},x_{s})+\int _{x_{0}}^{x_{s}}\lambda _{s}(x^{\prime})L_{s}(x^{\prime},\omega _{0})T(x^{\prime},x_{s})dx^{\prime}\nonumber \\ & =L_{0}(x_{0},\omega _{0})T(x_{0},x_{s})\nonumber \\ & \quad+\underbrace{\int _{x_{0}}^{x_{s}}\lambda _{s}(x^{\prime})L_{ss}(x^{\prime},\omega _{0})T(x^{\prime},x_{s})dx^{\prime}}_{direct\ illumination}\nonumber \\ & \quad+\underbrace{\int _{x_{0}}^{x_{s}}\lambda _{s}(x^{\prime})L_{ms}(x^{\prime},\omega _{0})T(x^{\prime},x_{s})dx^{\prime}}_{indirect\ illumination} \end{aligned}$$

where $L_{0}(x_{0}, \omega _{0})$, $T(x_{i},x_{j})$ and $L_{s}(x^{\prime}, \omega _{0})$ are described by Eq. 1. $L_{ss}(x^{\prime},\omega _{0})$ and $L_{ms}(x^{\prime},\omega _{0})$ are given by Eq. 2. This means that the direct and indirect illuminations are rendered separately. For the integral of indirect illumination, the incoming radiance $L_{ms}$ is evaluated using the VPM, which is detailed in Indirect illumination subsection.

### Preliminary: volumetric illumination model

**Fig. 2:**
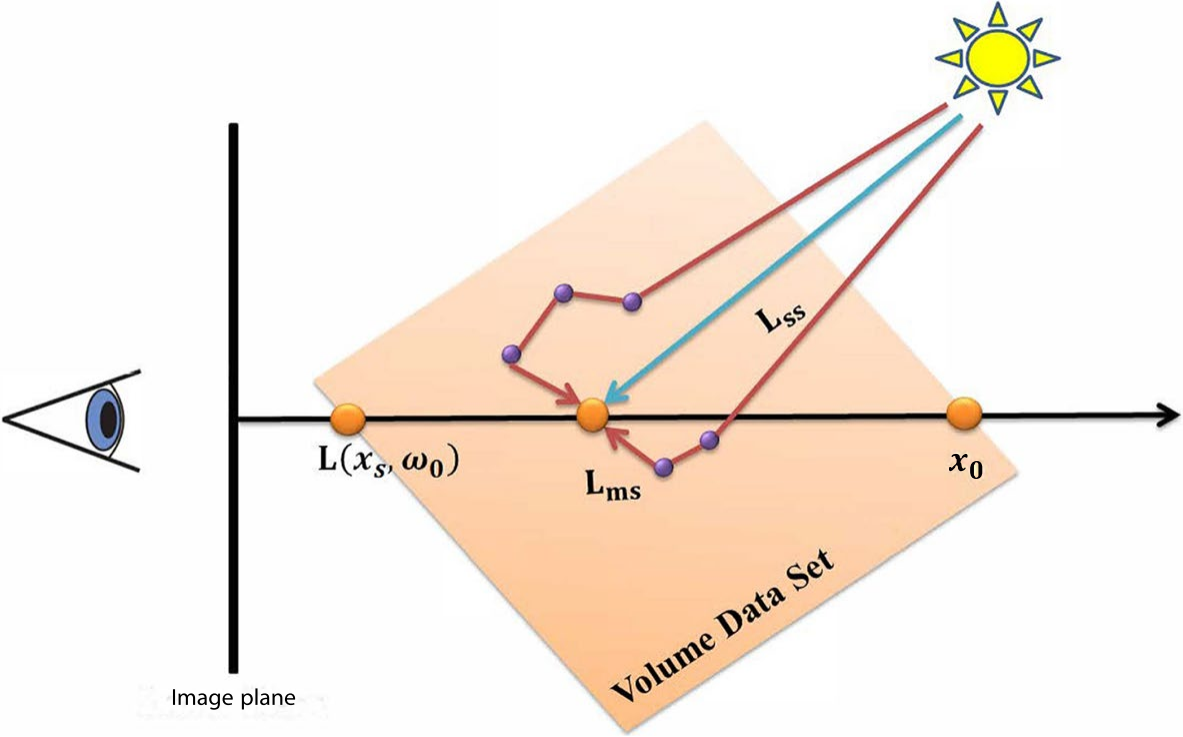
Diagram of the radiance transport in volume media. The SS effect is generated by scattering at one location towards the eye, represented as the blue line. MS covers more than one rebound before arriving at the eye, represented here using red lines

An overview of the radiance transfer estimation is presented in this subsection. According to ref. [36], photons are influenced by the emission, in-scattering, absorption, and out-scattering in the participating media volume. Radiance transport in the medium is illustrated in Fig. 2.

Given a sample $x_s$, along ray direction $\omega _0$, the radiance reaching $x_s$ is described following:

2 $$\begin{aligned} L(x_{s},\omega _{0}) & =L_{0}(x_{0},\omega _{0})T(x_{0},x_{s}) \nonumber \\ & \quad +\int _{x_{0}}^{x_{s}}\left[ \lambda _{a} (x^{\prime})L_{e}(x^{\prime},\omega _{0})+\lambda _{s}(x^{\prime})L_{s}(x^{\prime},\omega _{0})\right] T(x^{\prime},x_{s})dx^{\prime} \end{aligned}$$

where $L_{0}(x_{0},\omega _{0})$ represents the background energy entering the participant’s volume. $\lambda _{a}$ denotes the absorption coefficient and $\lambda _{s}$ denotes the scattering coefficient. $L_{e}(x^{\prime}, \omega _{0})$ denotes the radiance of a sample $x^{\prime}$ emitted and absorbed in the direction $\omega _{0}$ in the volume. $L_{s}(x^{\prime},\omega _{0})$ represents the radiance at position $x^{\prime}$ scattered in direction $\omega _{0}$. In addition, $T(x_{i},x_{j})$ describes the transmittances between samples $x_i$ and $x_j$, which are calculated according to $T(x_{i},x_{j})=e^{-\int _{x_{i}}^{x_{j}}\tau _{t}dt}$. Correspondingly, $\tau _{t}$ denotes extinction at time point *t*. To produce realistic rendering results, SS $L_{ss}(x^{\prime},\omega _{0})$ and MS $L_{ms}(x^{\prime},\omega _{0})$ must be considered.

3 $$\begin{aligned} L_{s}(x^{\prime},\omega _{0})=L_{ss}({x}^{\prime},\omega _{0})+L_{ms}({x}^{\prime},\omega _{0}) \end{aligned}$$

As shown in Fig. 2, $L_{ss}(x^{\prime},\omega _{0})$ denotes the reduced incident light $L_i$ that undergoes its 1st scattering interaction at $x^{\prime}$; this is regarded as direct illumination.

4 $$\begin{aligned} L_{ss}(x^{\prime},\omega _{0})=\int _{\Omega }s({x}^{\prime},{\omega }_{i},{\omega }_{0}){L}_{i}({x}^{\prime},{\omega }_{i})d{\omega }_{i} \end{aligned}$$

where $\omega$ depicts a set of different directions ${\omega }_{i}$ distributed on a local sphere centered at the sample $x^{\prime}$. Moreover, the material shading function is denoted as $s({x}^{\prime},{\omega }_{i},{\omega }_{0})$, which describes the amount of radiance originating from the direction $\omega _i$ and the scatterers in the direction ${\omega }_{0}$. A phase function is employed in the volume rendering to model the scattering behavior, which defines the probability that a photon will change its direction of movement by an angle $\theta$. The Henyey-Greenstein phase function, $p(\theta )=\frac{1-{g}^{2}}{{(1+{g}^{2}-2gcos\theta )}^{\frac{2}{3}}}$, is utilized to integrate phase functions, in which $g\epsilon [-1,1]$ is the anisotropy coefficient that produces back scattering, and isotropic and forward scattering. ${L}_{i}({x}^{\prime},{\omega }_{i})$ represents the attenuated incident radiance propagating through the volume in the case that there has only one light source at position $x_l$.

For MS, the photons are scattered several times before reaching the position $x^{\prime}$.

5 $$\begin{aligned} {L}_{ms}({x}^{\prime},{\omega }_{0})=\int _{\Omega }s({x}^{\prime},{\omega }_{i},{\omega }_{0}){L}({x}^{\prime},{\omega }_{i})d{\omega }_{i} \end{aligned}$$

Owing to the discrete nature of volumetric data, the volume-rendered integral must be approximated numerically. In practice, this is typically achieved by exploiting the Riemann sum.

### Pre-process

3D ultrasound images should be handled carefully before volume rendering to reduce the effect of speckle noise on the rendering results. To reduce the speckle noise, we employed the anisotropic diffusion filtering method designed by Huang et al. [37].

### Direct illumination

The reduced radiance from the light source serves as direct lighting for each voxel’s direct lighting, therefore VPM is not required at this stage. The direct illumination at scattering point $x'$, which is the solitary scattering contribution in our study, is calculated as follows:

1. Compute the engagement of light ${L}_{i}$ traveling through the light ray that is established by the connection between the light source and the scattering point $x^{\prime}$.
2. Sample a phase function to acquire the scattered ray direction. The interaction with the ray is used to calculate the contribution of the light traveling along it.
3. Compute ${L}_{ss}$ by combining scattering functions and the sampling light.

### Indirect illumination

This subsection discusses the main topic of addressing indirect illumination using VPM. Our method is a bidirectional path-tracing algorithm that combines a density estimation that samples eye and light paths (photons), and short eye paths are used to prevent expensive gathering. No sampled indirect eye paths were saved for the deterministic reflections.

#### Photon tracing

Creating photons for each basic light is the first step in the traditional VPM approach. We cast photons onto the volume’s boundary voxels to prevent photon wastage.

Millions of photons are released during this step. A GPU thread must be created for each photon released to track it, corresponding to the CUDA multi-thread programming model’s single instruction. Although this approach is straightforward, the threads are not completed simultaneously. A cap exists on the number of threads that a GPU can perform concurrently. Therefore, these threads are internally scheduled and run in batches according to GPU rules (For instance, the GPU in our experiments could support a maximum of 240 threads at most). The equal distribution approach is typically utilized for the released photons. Even with the robust independent concurrency of this method, it takes longer to track each photon than to emit the same number of photons because each photon has a unique path of refraction and reflection. Therefore, certain thread resources can be squandered, thereby prolonging the emission process.

Owing to the need for real-time interoperability, this technique should prevent resource wastage when attempting to increase emission efficiency. Thus, the photon emission tasks were co-completed by all threads in the manner examined in this research. To indicate the total number of photons released by all threads, we created the variable *Count* and set its initial value to 0. *Count* is increased by 1 units each time a thread emits a photon until it equals the number of photons released during the complete emission procedure. Consequently, the emission efficiency is improved, whereas the idle thread time is minimized.

#### Photon storing

We must track every photon emitted afterward and record its behavior in the scene. When a photon collides with a surface item, one of three processes can occur reflection, absorption, or transmission. The fate of a photon is typically determined using the Russian roulette method [29]. The effect that will occur and how it will affect the rendering outcome is determined by the material properties of the collision surface. Each route of the photon can be repeatedly stored, and information on how the photon is absorbed can be obtained. To save each photon, it is crucial to record the collision position, incidence direction, incident energy, and other information. In traditional VPM methods, photons are stored in KD tree data structures, which require considerable storage space; therefore, the last gathering process becomes time-consuming. To minimize these artifacts, we stored the photons in a volume texture.

#### Photon rendering

The major contributions of this study are discussed in this subsection. We acquired an indirect illumination volume once the photons were traced and stored, which were rendered using a ray-casting algorithm. Because the volume is overly small to be sampled, the conventional photon-mapping method employs ray marching for rendering, which is inappropriate for ultrasound images. For a sample along the ray, we have:

6 $$\begin{aligned} {L}_{{x}_{n}}=\sum \limits _{j=1}^{n}\prod \limits _{i=j}^{n-1}T\left( {x}_{i},{x}_{i+1} \right) \int _{{x}_{j-1}}^{{x}_{j}}T\left( {x}^{\prime},{x}_{j} \right) {\sigma }_{s}\left( {x}^{\prime} \right) {L}_{ms}\left( {x}^{\prime} \right) d{x}^{\prime}, j\ne n \end{aligned}$$

where ${x}_{j}$ denotes the j-th sample along the ray. If $j=n$, then $\prod \nolimits _{i=j}^{n-1}T\left( {x}_{i},{x}_{i+1} \right) =1$, $L_{ms}$ was calculated using density estimation of the photon map. The indirect illumination volume stores the flux of each photon, as previously mentioned. The flux and radiance have the following relationships:

7 $$\begin{aligned} L\left( {x}^{\prime},{\omega }_{0} \right) =\frac{{d}^{2}\Phi \left( {x}^{\prime},{\omega }_{0}\right) }{{\sigma }_{s}\left( {x}^{\prime} \right) d{\omega }_{0}dV} \end{aligned}$$

where *dV* is a volume element, ${d}^{2} \Phi ({x}^{\prime}, {\omega }_{0})$ is the photon flux ${x}^{\prime}$ in direction ${\omega }_{0}$. Using the destiny estimate, we can then calculate the MS radiances. The radiance of a photon is calculated using the spherically dispersed photon energy in its neighborhood [38, 39], which is a standard strategy for conventional photon density estimation. A normalized symmetric density estimation kernel was employed. This kernel assumes a planar surface about the estimates and overlooks photon density changes. A hierarchical data structure is required. In our approach, photons are stored using a volume texture, and the indirect illumination radiance is rapidly calculated using a new destiny estimation method. Direct illumination was not considered in this step. Next, the in-scattering radiance was calculated.

##### Conventional radiance estimation

**Fig. 3:**
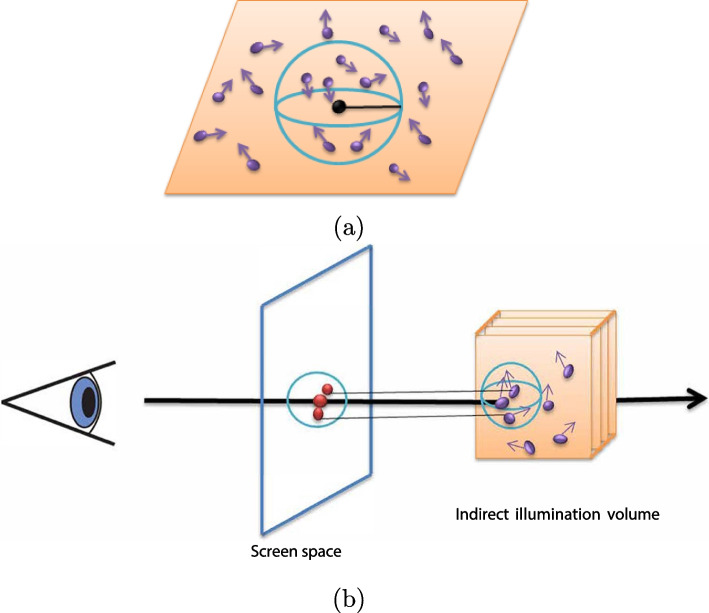
Destiny estimation. **a** is the conventional radiance estimation and **b** is the estimation in screen space

The inscattered radiance of each sample point along the beam is typically estimated using a photon map. The *n* closest photons are employed to estimate the radiance through a spherical kernel with radius *r* [29], as shown in Fig. 3a.

8 $$\begin{aligned} L_{ms}(x,\omega _{0}) & =\int _{\Omega }s({x}^{\prime},{\omega }_{i},{\omega }_{0})\frac{{d}^{2}\Delta \Phi ({x}^{\prime},{\omega }_{i})}{dV}\nonumber \\ & \approx \sum \limits _{i=1}^{n}s({x}^{\prime},{\omega }_{i},{\omega }_{0})\frac{\Delta \Phi ({x}^{\prime},{\omega }_{i})}{dV}\nonumber \\ & \approx \sum \limits _{i=1}^{n}s({x}^{\prime},{\omega }_{i},{\omega }_{0})\frac{\Delta \Phi ({x}^{\prime},{\omega }_{i})}{\frac{4}{3}\pi {r}^{3}} \end{aligned}$$

where $\Delta \Phi ({x}^{\prime},{\omega }_{i})$ is flux of the photon ${x}^{\prime}$; along the direction ${\omega }_{i}$, $s({x}^{\prime},{\omega }_{i},{\omega }_{0})$ denotes the material shading function.

##### Radiance estimation in screen space

Photon mapping may be seen as a problem in density estimation, as previously discussed. The number of photon hits can be used to represent the radiance of the samples. Thus, Eq. 5 can be represented as follows:

9 $$\begin{aligned} L_{ms}(x,\omega _{0})=\sum \limits _{i=1}^{n}s({x}^{\prime},{\omega }_{i},{\omega }_{0})\Delta \Phi ({x}^{\prime},{\omega }_{i})k({x}^{\prime}-{x}) \end{aligned}$$

where $k({x}_{i}-{x}^{\prime})$ is a low-pass filter. Nevertheless, the calculation time remains too lengthy to meet real-time requirements. Motivated by splatting [40, 41], a dual method for destination estimation portraying each photon as a tiny disc, one can compute the influence of the photon on the radiance evaluation. The photon energy, weighted by the kernel function, determines the value of each impact point. Because the sampling step in ray casting is considerably small and photons can be observed sequentially, we calculated MS effects in the screen space (as illustrated in Fig. 3b) rather than conventional radiance estimation. We projected rays from the image screen to the indirect illumination volume, rather than splitting photons onto the image screen and then sampling them.

Light traverses farther through the volume due to light diffusion from indirect lighting in dense materials, surpassing the distance expected if only direct attenuation were considered. Translucency implies that light blurs as it passes through the medium because of scattering. Gaussian blur was used to calculate the destiny estimation in two-dimensional (2D) image space. An image-space effect known as Gaussian blur was used to produce a slightly blurred version of the original image. Gaussian blur is often used as an N$\times$N-tap convolution filter to reduce the image noise by weighting the pixels inside its footprint based on the following Gaussian function:

10 $$\begin{aligned} G\left( r \right) =\frac{1}{\sqrt{2\pi \delta ^{2}}}e^{\frac{-r^{2}}{2\delta ^{2}}} \end{aligned}$$

where $\delta$ is the value of the standard deviation. When this value increased, the generated fuzzy effect became more prominent. Additionally, *r* is the blur radius, that is, the distance between the boundary and center of the Gaussian blur window. When the blur radius increases, more pixels are enclosed, producing a more blurred effect. However, the edges of an image may be missing if adopting the Gaussian blur in texture images. The separability of a Gaussian function can be used to resolve these issues.

Using the separability of Gaussian functions, 2D Gaussian functions were transformed into a one-dimensional matrix. Using the fragment shader implementation, splitting the Gaussian filter into horizontal and vertical components continues to yield accurate results. Consequently, there are two N-tap filters, with the second requiring a separate rendering pass. In this case, the time complexity is $O(n\times M\times N)+O(m\times M\times N)$ whereas the previous time complexity without separation is $O(n\times m \times M\times N)$, where *m* and *n* represent the dimensions of the Gaussian matrix and *M* and *N* are the dimensions of the 2D image. The time complexity was less than that of the previous approach by an order of magnitude. In our method, we chose the dimensions of the Gaussian matrix to be $3\times 3$.

### HDR

Using our approach, volume rendering results with a wide range of light intensities were obtained. However, the ultrasound display’s constrained field-of-view of the ultrasound display may be insufficient to convey the outcomes of considerably accurate volume rendering. Therefore, we introduced a postprocessing step to correct images with a wide range of intensities using HDR techniques that are frequently employed in digital photography. Compared with conventional low-dynamic-range photography approaches, HDR techniques enable users to tackle images with larger luminance dynamic ranges. One of the most widely used HDR techniques for preserving the local contrast between features is the tone-mapping approach [42], which maps a high range of luminance in HDR photos to standard devices that have lower dynamic ranges. The HDR tone-mapping algorithm is a global operator. Global operators are spatially uniform because they are nonlinear functions that depend on luminance or other global factors. In other words, regardless of the values of the surrounding pixels, the tone mapping is the same for each pixel in the image.

### User evaluation

Lindemann and Ropinski [43] proposed that a user study could evaluate the effectiveness of advanced illumination. To analyze whether the visual perception was improved by our illumination model, we also conducted a user study. The primary visual enhancement involved using scattering to approximate the skin and enhance depth perception. Based on this, two tests were performed on each participant to evaluate the usefulness of our method. Thirteen participants (ages 25-36 with an average age of 30 years) were recruited. Most participants were students or members of the Chinese Academy of Sciences and Sichuan University affiliated with the Department of Obstetrics and Gynecology. All participants had normal or corrected-to-normal vision and four wore glasses. As our 3D ultrasound rendering method is mainly focused on the diagnosis and communication between pregnant women and doctors, we selected more females to attend our evaluation. Eleven females and two males were selected, and six women became pregnant.

**Fig. 4:**
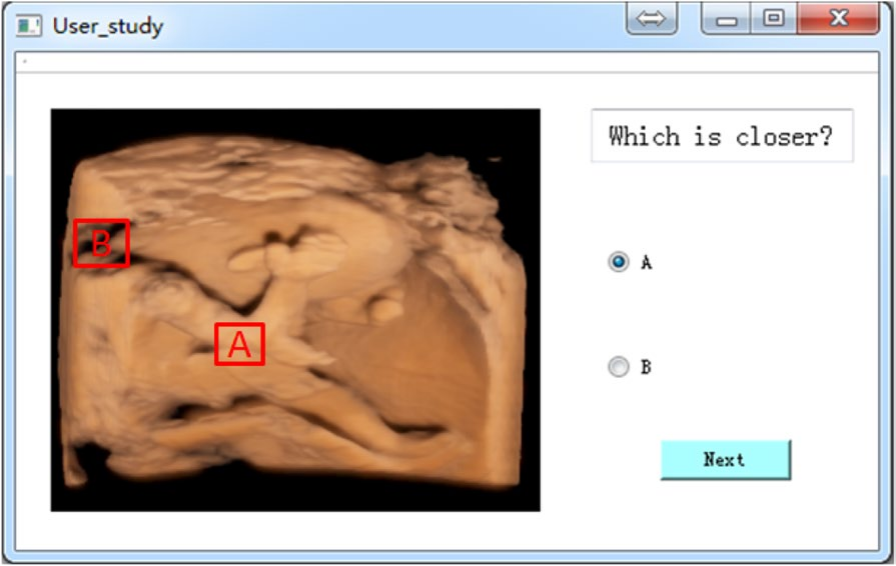
A snapshot of the test window

Our test consisted of two parts: a skin-rendering comparison test and a depth comparison test. In addition, each test began with a straightforward illustration to acquaint participants with the format. Figure 4 presents a snapshot of the test window. The rendered images are displayed in the left-hand window. To reduce side effects, the images used in these two tests were randomly displayed. Figure 4 shows only the depth comparison test. The participants were asked to select which part was similar to the real fetus examination or which part was closer to the user. All participants underwent a short training session before the test.

## Results

In this section, the rendering results are summarized. The effectiveness of our proposed rendering technique was assessed by using retrospective ultrasound data of several actual fetuses at various stages of pregnancy. To implement our technique, C/C++, OpenGL, and CUDA were used. A graphical user interface was created using the Qt. All the results were produced on a PC with an Intel E5 processor 8GB of memory and a graphics card.

### Graphical user interface

**Fig. 5:**
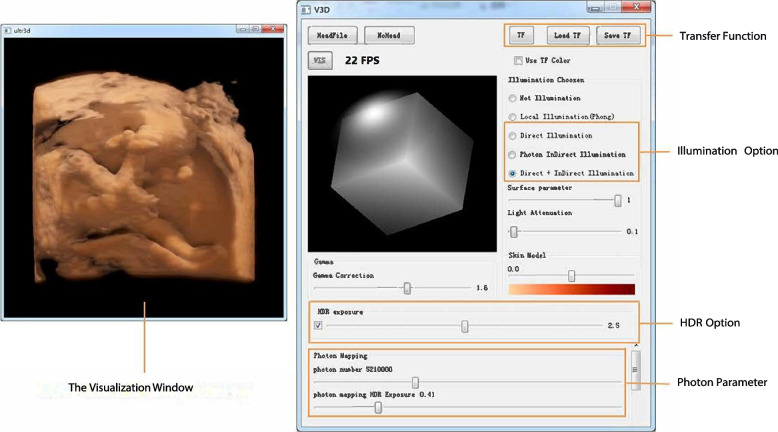
The user interface of realistic rendering system

The user interface of our realistic rendering software is shown in Fig. 5. The rendering outcome is shown in a separate window on the left side of the screen, which can be freely resized. All system parameters are displayed in a separate window on the right side of the screen, referred to as the operation window. Our approach uses a two-step rendering process with direct and indirect illumination. We offer an illumination choice and display the scattering factor to ensure that users can easily see the results of the two processes. If users aim to adjust the number of photons, the photon mapping parameters are displayed at the bottom of the operating window.

### Example of fetal ultrasound

**Fig. 6:**
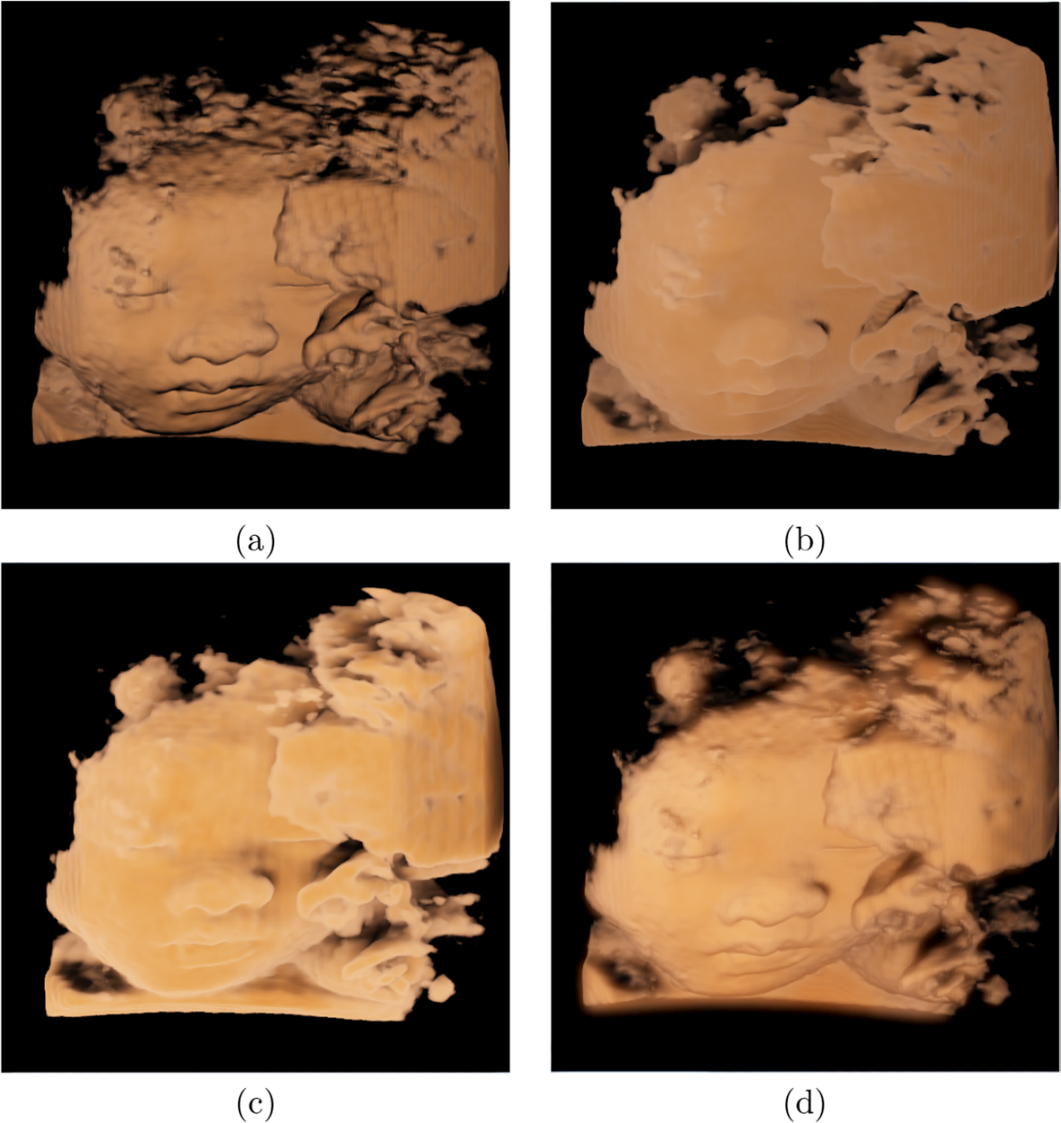
Rendering results of fetal data 1 with different techniques. **a** Results of using local illumination; **b** Results of using half-angle slicing; **c** Results of our method using only direct illumination, and **d** Results of our method employing both direct illumination and indirect illumination. The images used are the same as those in https://ieeexplore.ieee.org/document/8241654/

**Fig. 7:**
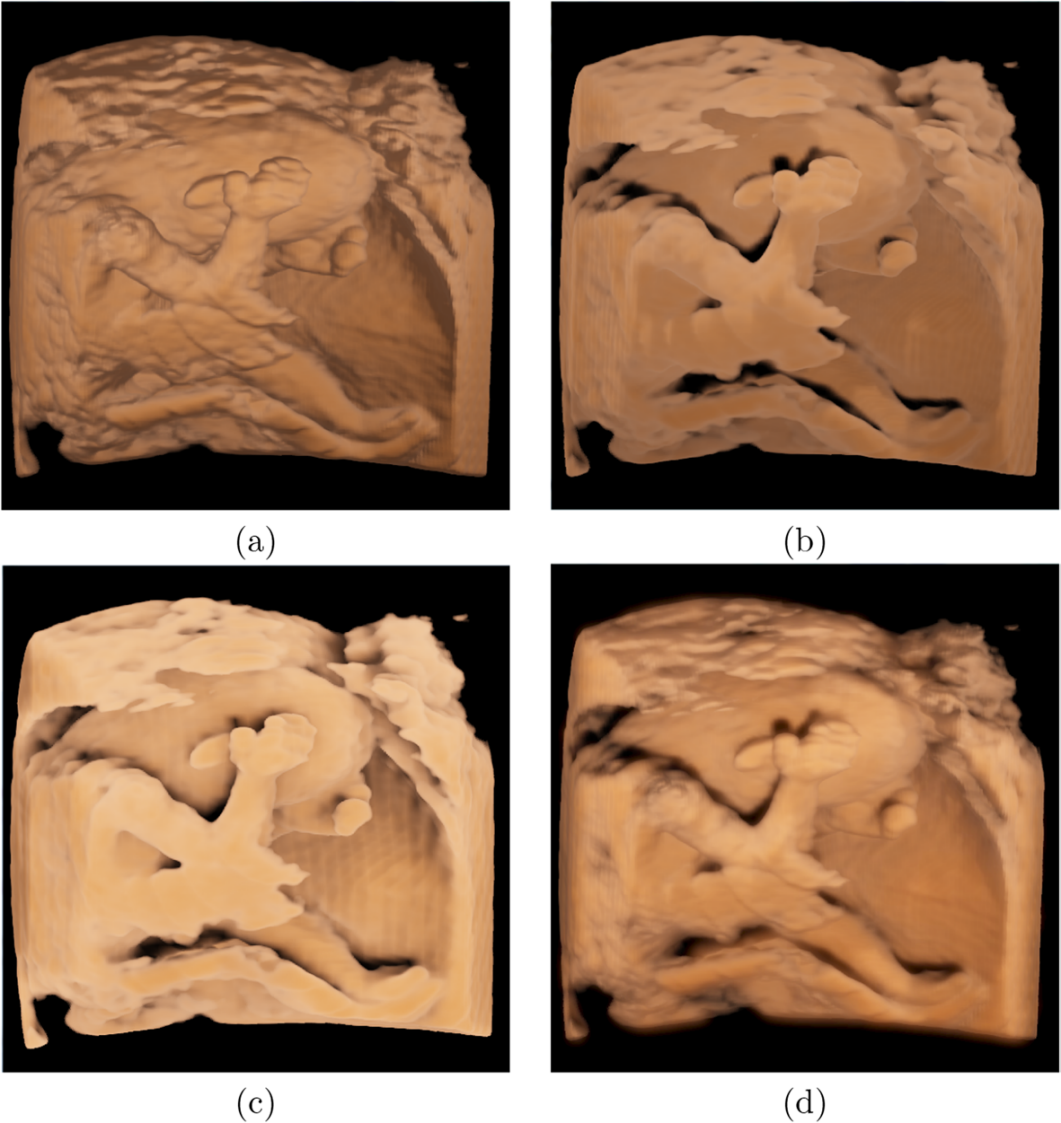
Rendering results of fetal data two with different techniques. **a** Results of using local illumination; **b** Results of using half-angle slicing; **c** results of our method using only direct illumination; and **d** Results of our method employing both direct illumination and indirect illumination

To assess the accuracy of our method, we display the rendering results of two fetal datasets using our method and the results elicited with local illumination that can be treated as ground truths in Figs. 6 and 7. The positions of the light sources and transfer functions, among other factors, were set to be the same in both cases. Figures 6a and 7a show the rendered results of fetuses 1 and 2 using the local illumination method [1]. The most popular approach for simulating global illumination in ultrasound volume rendering is half-angle slice rendering. Additionally, this rendering technique was also applied to the GE Voluson machine [44, 45]. Figures 6b and 7b show the results obtained using this method on fetuses 1 and 2. To demonstrate our two-step algorithm, Figs. 6c and 7c illustrate the rendered results that adopt only direct illumination. These findings show that our system can simulate the fetus’ surface accurately; however, the wood grain can be observed because scattering across voxels was not considered. The rendered results obtained using the global illumination method are as follows: Figs. 6d and 7d show the direct and indirect illumination, respectively.

**Fig. 8:**
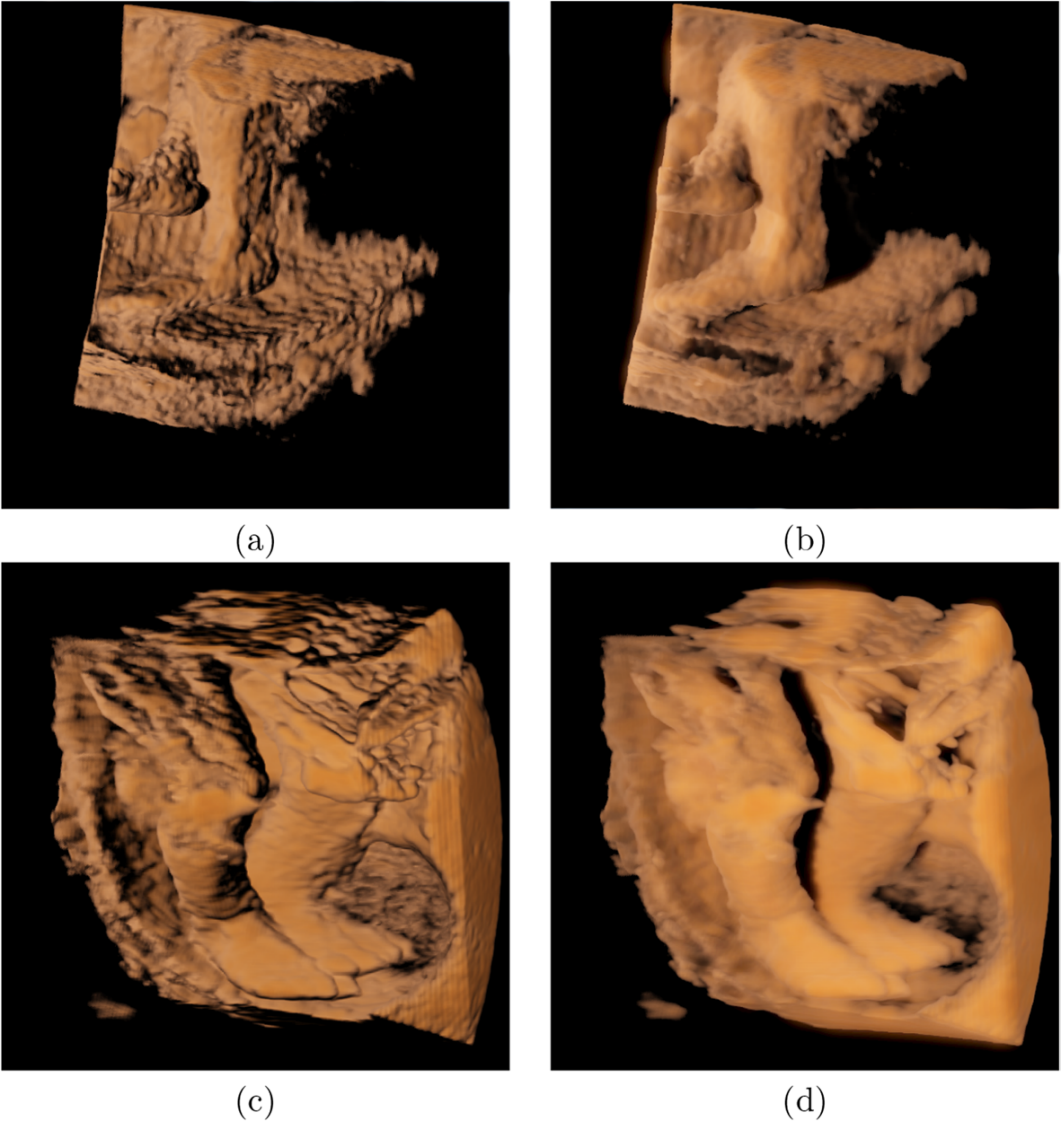
Rendering results using local illumination are shown in the first column and the results of our method are shown in the second column

Physicians may monitor the development of the legs and feet. The visualizations of fetus data 3 and 4 using our rendering system are shown in Fig. 8. Figures 8a and c show the results with local illumination, where the skin of the fetus appears unrealistic, and the depth orders of the two legs are fuzzy. Figures 8b and d present the results using our global illumination. From Figs. 8b and d, we observe that the right foot is in front of the left foot and that the amniotic membranes and the left foot can be separated.

### Interactivity

**Fig. 9:**
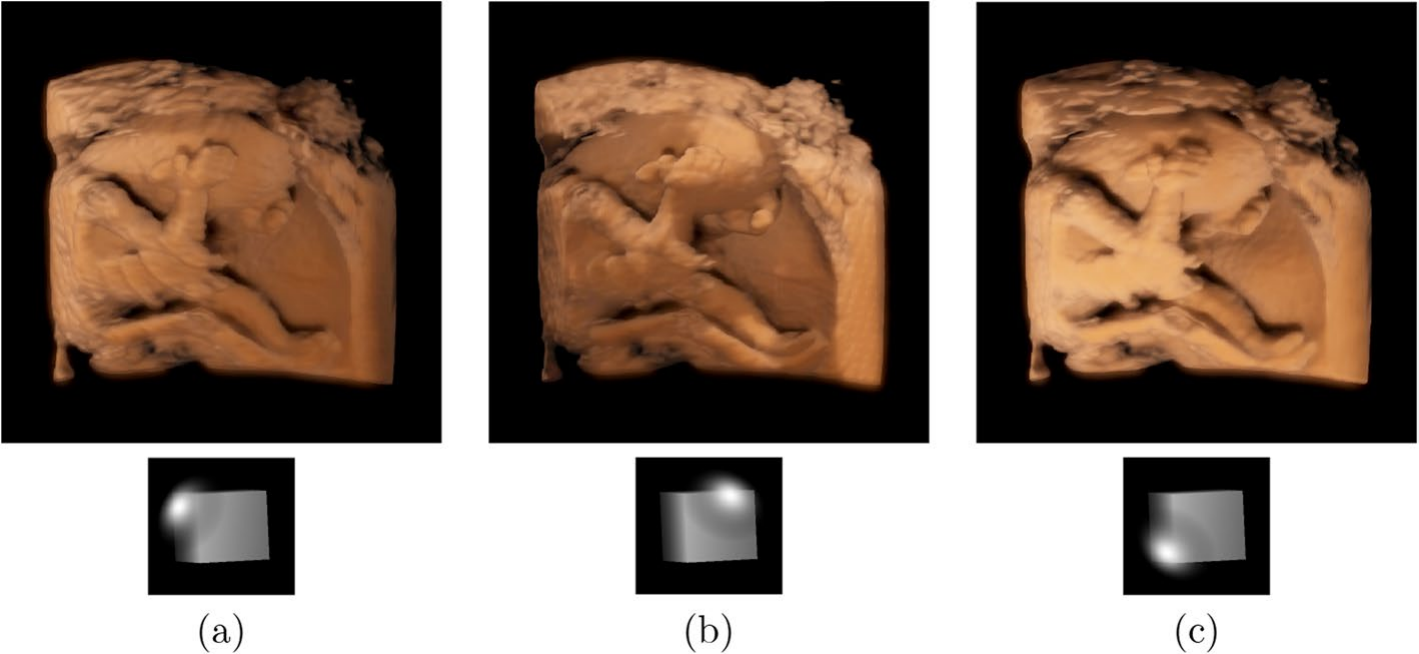
Rendering results with different light positions

As our system is capable of real-time acquisition, our illumination model allows users to change the transfer functions and position of the virtual light source. The actual position of the light source is important for clinicians, who may want to adjust it to identify fetal organs. The rendered results for fetus 2 at different light positions are shown in Fig. 9. Figure 9a presents the results when the light is in the upper-left corner. This position can help physicians to observe the nape more clearly. Figure 9b shows the results when the light was in the upper right, whereby the shape perception of the hands and ears could be enhanced to help physicians observe them more clearly. Figure 9c shows the results when the light was in the bottom-left part and the depth perception of the legs and feet was enhanced.

**Fig. 10:**
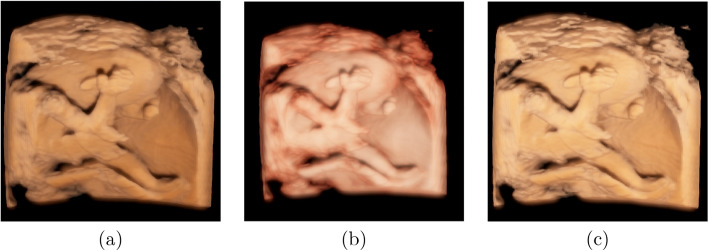
Rendering results with different transfer functions

The transfer function is also an important parameter for volume rendering, and physicians can observe different tissues by changing the transfer functions, while different rendering colors can provide pregnant women with additional experience. Our method can support physicians in changing the transfer functions in real-time. Figure 10 shows the rendering results for the different transfer functions. Figures 10b and c show the different color and opacity transfer functions.

### Performance

Four datasets were evaluated using our rendering system, all of which were rendered in real-time. Table 1 shows the performance by frames per second (FPS), where exposure is the luminance of each photon, samples denote the number of samples in the rendering process, and FPS is the measurement of the rendering speed. The number of photons and the amount of data both significantly affect the effectiveness of our strategy.

**Table 1:** Performance assessment for the fetal data

Name	Sample	Resolution	Exposure	Number of photon	Render
Fetus 1	800	$289\times 212\times 266$	0.42	6.2	17
Fetus 2	800	$183\times 115\times 126$	0.41	5.2	22
Fetus 3	800	$209\times 175\times 209$	0.38	4.1	18
Fetus 4	800	$196\times 128\times 122$	0.41	3.5	20

**Fig. 11:**
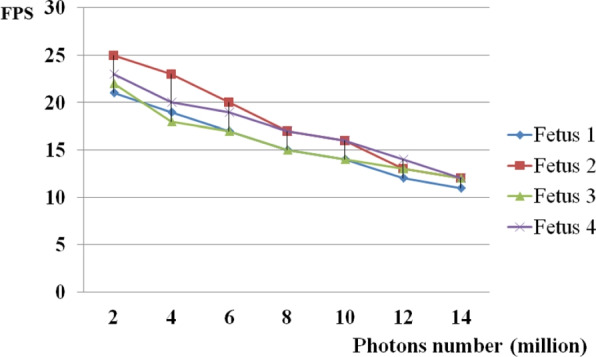
Performance of datasets with different photons numbers

A more realistic scattering effect can be produced with more photons; however, the pace of rendering is slower. Figure 11 shows the performance of all fetal datasets in the FPS with different photons.

### User evaluation results

As mentioned previously, the outcomes of the two tests, namely, the skin rendering comparison test and the depth comparison test, are included in the evaluation results.

#### The skin rendering comparison test

Twenty-two pairs of images were rendered separately using our illumination model and local illumination. For each pair of rendered images, other parameters (e.g., transfer functions, camera, and light position) were kept constant. The participants were required to select the image they felt was closer to the actual fetal skin and more visually pleasing from each pair of images.

#### The depth comparison test

Thirty-six rendered images were shown to each participant and three regions were marked for each image. These images were rendered using the proposed method or local illumination. The participants clicked on the region they considered closer to the viewer. The correction and selection times of the participants were recorded to measure their responses.

**Fig. 12:**
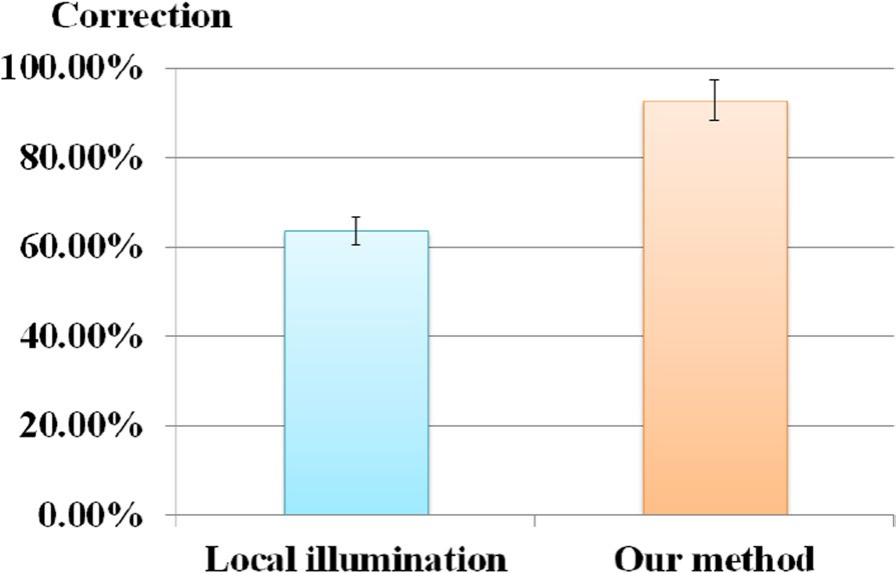
Results of the depth comparison tests: Depth decision accuracy

Skin rendering comparison testing showed that, in $93.6\%$ of the instances, the subjects thought our model was more realistic. The results of the depth-comparison test are shown in Fig. 12. As indicated in Fig. 12, $92.8 \%$ of the cases perceived the correct depth decisions in the rendered images when our method was used, whereas only $63.6 \%$ of the cases noted the correct depth when local illumination was used. The relative performance of local illumination compared to our method was $63.8/92.8=0.69$. Finally, as both of these results denote the frequency distribution of events, a t-test was employed to analyze the results, and we found that the increase in accuracy was significant (*p*-value < 0.01). We ran a t-test on both of these results and discovered that our method considerably (*p*-value < 0.01) enhanced the accuracy and speed.

## Discussion

As mentioned in Method section, most previous studies have used a local illumination model to simulate global illumination, and half-angle slicing has also been proposed and used in some ultrasound machines. Figures 6 and 7 show the comparison images of the rendered results of our method against the local illumination and half-angle slicing methods. The comparison shows that the images rendered using local illumination are unrealistic and have no depth perception. The half-angle slicing method can simulate realistic skin; however, it requires dividing the volume into slices, resulting in wood-grain effects in the rendered output. In addition, this method only simulates the propagation of light from the front to the rear, whereas our method uses random propagation, which is closer to real global illumination. As shown in Figs. 6c and d and 7c and d, owing to the consideration of the dispersion effects, our method can reflect the fetal volume more accurately. The images show that our volume-rendering method can improve the perception of the nose and lower lip, which are enlarged spatially after rendering. This improvement can help physicians diagnose whether the face of a fetus is abnormal. Furthermore, a soft shadow can be produced when the scattering action in the medium is considered, which reduces the appearance of the wood grains. Additionally, our method can enhance the depth information, as shown in Figs. 8b and d. In these two images, the right and left legs can be distinguished.

Compared with other studies that also use photon mapping to simulate global illumination, our method can achieve real-time imaging by rendering direct and indirect illumination separately and by computing the radiance of indirect illumination in the screen space. Table 1 shows that for large datasets, such as fetus 1, with a volume size of $289\times 212\times 266$, our method also achieves real-time rendering. Figure 11 shows that even when the number of photons increases to ten million, our method achieves real-time results. The photon number is an important rendering parameter because the exposure of each photon can influence the brightness of the rendered results. We must cast more photons for large datasets; otherwise, some of the rendered results may be unrealistic when the photon content is low.

Because our method must cast large photons to ensure adequate rendering quality, it must be applied to machines with a GPU card. This is the current limitation of the proposed approach for real medical applications.

3D ultrasound is primarily used to detect surface abnormalities. It is difficult to visualize the inner (or deep) structures because of their relatively low image quality. However, if we design a good transfer function for 3D ultrasound rendering, we can observe the inner (or deep) structures. Our work focuses on the global illumination model of 3D ultrasound rendering, and the transfer function design is beyond the scope of this study. In future work, we will consider combining good transfer functions with the proposed illumination model. The combination of Doppler and 3D ultrasonography is a promising approach, and we will explore the potential integration of Doppler data into our future visualizations.

## Conclusions

In this study, global illumination was used to develop a realistic and timely volume-rendering technique for 3D ultrasound fetal examinations. To accelerate rendering, direct and indirect illumination are saved in texture volumes and rendered individually. A novel radiance estimation approach was designed because conventional estimation requires complicated data structures and cannot provide timely interactions for 3D ultrasound volume rendering. More crucially, the VPM algorithm was employed to estimate indirect illumination. The results reveal that our proposed system can produce more realistic rendering effects, and user evaluation proves that our system successfully improves visual perception.

## Data Availability

The datasets analyzed in the current study are not publicly available but are available from the corresponding author on reasonable request.

## Abbreviations

HDR: High dynamic range
3D: Three-dimensional
AO: Ambient occlusion
SS: Single scattering
MS: Multiple scattering
VPM: Volumetric photon mapping
KD: K-dimensional
FPS: Frames per second
2D: Two-dimensional
